# Getting through the voices

**DOI:** 10.1038/s41537-022-00298-w

**Published:** 2022-10-18

**Authors:** Jason Jepson

**Affiliations:** Students with Psychosis, Myrtle Beach, SC USA

**Keywords:** Diseases, Schizophrenia

Because I have a severe mental illness, I sometimes hear voices. I have come to realize that this is a symptom of schizoaffective disorder which is my diagnosis. People like me who have the same, or a similar brain disease, may hear voices which seem to want to take over our lives. This symptom is most difficult for me to manage between the hours of 6 pm and 9 pm.

These voices are almost always negative in nature and can be very annoying if I get caught up in them. They are cutting, demeaning, and seem to have as their goal to defeat me. These wearisome voices are telling my neighbors terrible things about me.

When I actually hear my neighbor’s voice, it is loud, and I recognize her voice as real. I also hear footsteps, and dogs barking. I know this is all real. It is hard to explain, but I hear a difference between the human voices in the morning and the voices in my head the night before. The sound of my neighbors, voices are genuinely concerning to me. There have been car break-ins in my apartment’s parking lot, and I fear my neighbors think I am the one who is doing it because I have schizophrenia.

The voices are muffled similar to whispers. Just recently I heard a voice telling my neighbors that I have schizophrenia, and that is the reason I stay to myself. The voice sounded exactly like my neighbor, but I had never told her that I have schizophrenia, so I know the voice I heard is the result of my brain disease not part of my reality.

The voices that I hear often belong to acquaintances and friends from my past life, such as an ex-girlfriend. I may not have spoken to them in years. In my head, they say they do not want to talk to me because my other voices would bother my friends and distract them from their jobs.

Sometimes it seems like my voices try to make me forget things, like listening to my live online church service. I know this is not really going on, however, fearing I might oversleep and miss the service, I decided to set my alarm clock. The church service is at 11 am, so I set my alarm for 10 am.

Often the voices distract me to the point of causing me to be forgetful. I fear that I will forget to pull up my zipper after using a public restroom. The voices laugh and say they won because they were able to embarrass me. Sometimes when I am driving, I forget where I am going. Because of my forgetfulness, I always make a grocery list before I go to the store.

Even though I hear voices that can interrupt my life, I take my medication on a regular basis, and I have never missed a mental health appointment. Even so, I deal with these voices most every night between the hours of six and eight o’clock. Of course, I know that hearing voices is a symptom of schizophrenia. I get through those evening hours by trying not to dwell on the voices or to get swept up in conversation with them.

Between the hours of 6:00 PM and 8:00 PM, I try to distract my mind, so I do not hear the voices. I watch a TV show or movie. I also listen to music. Planning ahead helps me alleviate the stress that comes from hearing voices. During these times, I am glad I live alone so I can do whatever I need to do to distract myself.

I am a grown man and I like having my own space to do what I want, even with schizophrenia as my roommate. I am prescribed a sleeping aid, or else I might be awake all night with my racing thoughts and delusions.

It is very satisfying to get through a night of excessive voices. I share what I learn to different schizophrenia support groups on different social networks. It helps to realize I am not the only one going through these challenges. A beautiful new day is always near. I love waking up in the morning to silence.

